# Post-translational modifications of PML: consequences and implications

**DOI:** 10.3389/fonc.2012.00210

**Published:** 2013-01-04

**Authors:** Xiwen Cheng, Hung-Ying Kao

**Affiliations:** ^1^Department of Biochemistry, School of Medicine, Case Western Reserve UniversityCleveland, OH, USA; ^2^Comprehensive Cancer Center, Case Western Reserve UniversityCleveland, OH, USA; ^3^University Hospital of Cleveland, Case Western Reserve UniversityCleveland, OH, USA

**Keywords:** PML, post-translational modification, sumo, SUMOylation, phosphorylation, acetylation, review

## Abstract

The tumor suppressor promyelocytic leukemia protein (PML) predominantly resides in a structurally distinct sub-nuclear domain called PML nuclear bodies. Emerging evidences indicated that PML actively participates in many aspects of cellular processes, but the molecular mechanisms underlying PML regulation in response to stress and environmental cues are not complete. Post-translational modifications, such as SUMOylation, phosphorylation, acetylation, and ubiquitination of PML add a complex layer of regulation to the physiological function of PML. In this review, we discuss the fast-moving horizon of post-translational modifications targeting PML.

## INTRODUCTION

**FIGURE 1:**
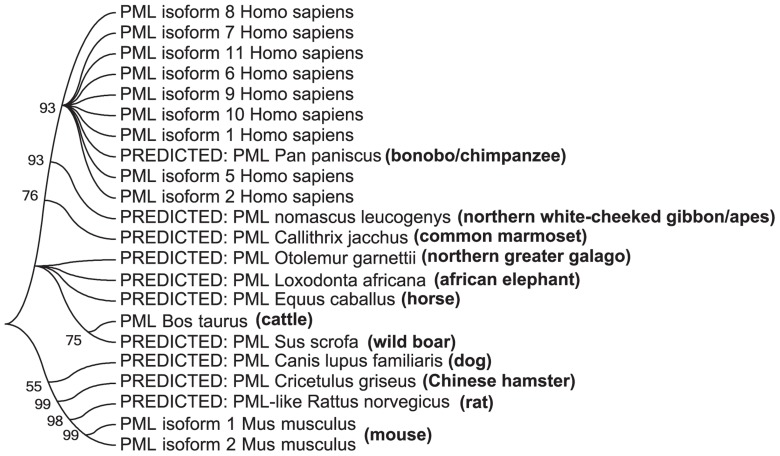
**Phylogenetic tree of PML proteins in mammals**. The phylogenetic tree was generated using the maximum likelihood method with amino acid substitutions in the Jones–Taylor–Thornton model at a uniform rate. The bootstrap score is labeled at the branches.

**FIGURE 2:**
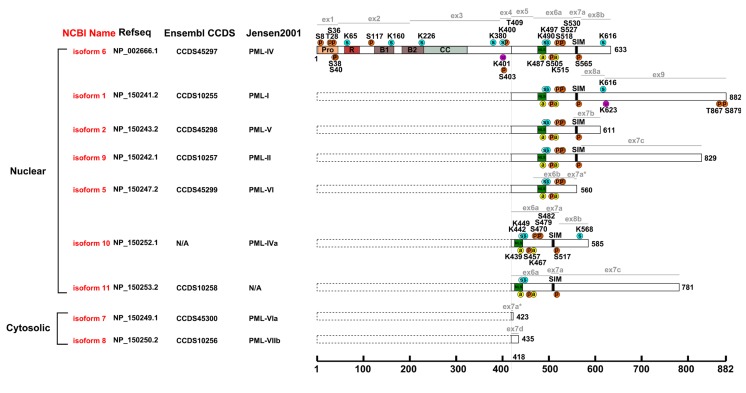
**Post-translational modifications of human PML**. Alignment of human PML isoforms with post-translational modifications annotated. Pro, proline-rich region; R, RING domain; B1 and B2, B-Box domains; CC, predicted coiled coil region; circled p, phosphorylation site; circled s, SUMOylation site; circled u, ubiquitination site; circled a, acetylation site; ex1–9 and lines in grey, exon boundaries; dotted box, the consensus sequence for all PML isoforms (1–418); isoforms 1-2 and 5-11 in red, NCBI nomenclature; Refseq, NCBI reference sequence; Ensembl CCDS (Consensus CDS); Jensen2001 (Nomenclature proposed by Jensen et al. (2001). The post-translational modifications are positioned using one-letter amino acid code and the position number in the corresponding isoform.

Promyelocytic leukemia protein (PML) is a tumor suppressor that was initially identified as a fusion partner of human retinoic acid receptor alpha (RARα) as a result of a chromosomal translocation found in the acute promyelocytic leukemia patients (APL; de Thé et al., 1991; Kakizuka et al., 1991). PML is expressed and conserved in all mammals (**Figure 1**). It is enriched in proteinaceous masses called PML nuclear bodies (NBs), which are visualized as spherical nuclear speckles (Ascoli and Maul, 1991; Daniel et al., 1993). Many proteins have been identified as PML interacting partners or components of the PML NBs. PML NBs are implicated in various cellular activities, including transcriptional regulation, cell cycle control, post-translational modification, anti-viral response, DNA damage response and repair, apoptosis, and metabolism (Kitamura et al., 2005; Van Damme et al., 2010; Kim et al., 2011; Carracedo et al., 2012; Cheng and Kao, 2012; Ito et al., 2012). There are nine experimentally verified isoforms in human according to the NCBI database (**Figure 2**), all of which have the N-terminal 418 amino acids in common. A nomenclature system of PML isoforms using roman numerals was proposed by Jensen et al. (2001). Although widely used by researchers, this nomenclature system, however, has not converged with the references used in common sequence databases of NCBI or Ensembl. Sometimes, ambiguous references were presented in the literature. In this review, we will refer to the PML isoforms using the names currently implemented by the NCBI database and annotate each isoform with names from other nomenclature systems (**Figure 2**). There is only limited information available on the function of most of the isoforms; although a growing body of evidence suggests that different isoforms may have specific functions. For example, PML isoform 2 is implicated in scaffolding PML NBs (Weidtkamp-Peters et al., 2008), while PML isoforms 1 and 9 are involved in the antiviral activity (Cuchet et al., 2011). PML isoform 6, the best-studied isoform, interacts and recruits p53 to PML NBs (Fogal et al., 2000). Unless otherwise specified, this review will summarize our understanding of the post-translational modifications using PML isoform 6 as a reference and focus on SUMOylation, phosphorylation, ubiquitination, and the newly identified acetylation. These modifications regulate the ability of PML to interact with various partners and confer stress- and signal-dependent regulation of PML or its binding proteins.

## SUMOylation OF PML

Human small ubiquitin-like modifiers (SUMOs) include three paralogs: SUMO1, SUMO2, and SUMO3. SUMO2 and SUMO3 share 95% sequence identity while SUMO1 is only 50% identical. Protein SUMOylation involves a three-enzyme cascade: a single sumo activation enzyme E1 dimer (SAE1/SAE2; Gong et al., 1999; Okuma et al., 1999), a sole E2 conjugating enzyme (UBC9; Desterro et al., 1997; Gong et al., 1997; Bernier-Villamor et al., 2002) and multiple substrate-specific E3 sumo ligases (Geiss-Friedlander and Melchior, 2007). SUMOylation regulates several aspects of a target protein including protein stability, sub-nuclear localization, transcriptional activity, and protein–protein interactions (Geiss-Friedlander and Melchior, 2007). SUMO2 and SUMO3 contain a lysine at position 11 (K11) that can be conjugated to themselves or with SUMO1 and usually form poly-SUMOylation chains. By contrast, SUMO1 does not contain K11 and is conjugated to its substrates once or marks the end of poly-SUMOylation chain.

**FIGURE 3:**
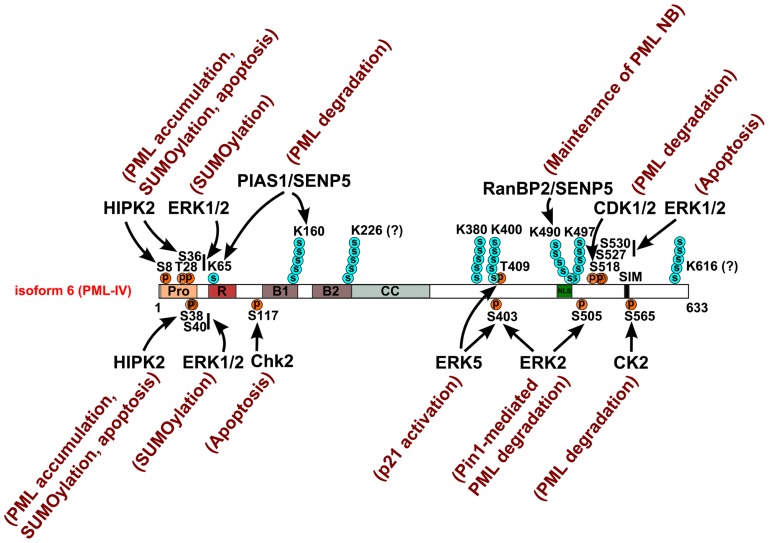
**Site-specific kinases, SUMO E3 and deconjugating enzymes that target PML**. The diagram depicts the modified residues in PML targeted by kinases, SUMO E3 ligases, or SUMO deconjugating enzyme. Arrows indicate the targeting site(s) of these enzymes. Poly-SUMO chains are observed at K160, K380, K400, K490, and K497 and tentatively at K226 and K616. K65 is modified by either SUMO1 or a poly-SUMO chain. The functional consequences of these post-translational modifications are annotated adjacent to the corresponding enzyme.

SUMO1 was initially identified as a PML interacting protein through a yeast-two hybrid screen (Boddy et al., 1996). This interaction requires a SUMO-interacting motif (SIM) at the C-terminus of PML (Lin et al., 2006; Shen et al., 2006). A body of evidence has demonstrated that PML is post-translationally conjugated to SUMO1 (Sternsdorf et al., 1997; Kamitani et al., 1998b; Muller et al., 1998) and SUMO2/3 (Kamitani et al., 1998b). Initial studies identified three canonical SUMOylation sites K65, K160, and K490 (Kamitani et al., 1998a) on PML. Additionally, later studies also suggested potential poly-SUMO conjugation sites at K226 and K616 (Vertegaal et al., 2006) and identified three poly-SUMO conjugation sites including K380, K400, and K497 in response to arsenic trioxide treatment (Galisson et al., 2011; **Figure 3**). By immunofluorescence microscopy, endogenous PML, and SUMO1 were found colocalized in PML NBs (Muller et al., 1998; Gao et al., 2008a). PML NBs are thought to be a nuclear depot where SUMOylation elicits its various roles through modulating PML or components of PML NBs.

SUMOylation is essential for maintaining proper PML NB structure and normal function. Although PML dimerization is the prerequisite for *de novo* PML NB assembly, SUMOylation of PML is required for the recruitment of components of PML NBs (Lallemand-Breitenbach and de The, 2010), the turnover and retention of PML in PML NBs (Weidtkamp-Peters et al., 2008), and the integrity of PML NBs (Shen et al., 2006). A few studies have examined the modification pattern of PML by specific SUMO isoforms. Mouse embryonic fibroblasts (MEFs) derived from SUMO1 knockout mice show reduced SUMOylation of PML by SUMO2/3 and marked decreases in the number of PML NBs compared to those in the wild type cells, suggesting that SUMO1 conjugation of PML is important to maintain the integrity of PML NBs (Evdokimov et al., 2008). SUMO3 conjugation at K160 also regulates nuclear localization of PML and PML NB formation (Fu et al., 2005; **Figure 3**). Additionally, viral protein LANA2 promotes SUMO2-conjugation of PML (Marcos-Villar et al., 2011). However, how this pattern originates and what mechanism determines the specificity by which SUMO1, -2, or -3 is conjugated to specific lysine residues is still largely unknown. SUMOylation of PML also regulates the localization of other components in PML NBs (Ishov et al., 1999; Li et al., 2000; Zhong et al., 2000). The SUMOylation moiety on PML and/or other components of PML NBs interact through SIMs present in these proteins (Takahashi et al., 2005; Lin et al., 2006). The ability of PML to interact with sumo-conjugated moieties of other PML NB components is necessary for PML NB formation (Shen et al., 2006). SUMOylation of PML exhibits a cell cycle-dependent pattern accumulation. It is elevated during interphase and declines during mitosis (Everett et al., 1999). Additionally, a specific form of PML NBs was observed in human neuronal intranuclear inclusion disease associated supraoptic neurons that exhibits as a single large proteinaceous inclusion body enriched with PML, SUMO1, and UBC9 (Takahashi-Fujigasaki et al., 2006; Villagra et al., 2006), raising a possibility that SUMOylation of PML functions in a tissue-specific manner.

SUMOylation of PML is a key regulator that controls PML stability in response to extracellular or intracellular stimuli. Arsenic trioxide (As_2_O_3_), a long-known promising therapeutic agent for treatment of APL, induces a group of slow-migrating PML species that were identified as SUMO-conjugated PML (Muller et al., 1998). Arsenic trioxide mediates PML degradation; however not until recently the molecular mechanism has been largely elucidated. Arsenic trioxide directly binds to the cysteine-rich zinc fingers in the RING finger, B-box and coiled coil (RBCC) domain of PML (**Figure 2**). As_2_O_3_ binding directs a conformational change of PML that promotes the interaction between PML and the SUMO2 conjugation E2 enzyme UBC9 (Zhang et al., 2010). Interestingly, such As_2_O_3_-induced PML SUMOylation is inhibited by treatment with the serine/threonine phosphatase inhibitor calyculin (Muller et al., 1998), suggesting that certain cellular phosphorylation event inhibits As_2_O_3_-induced PML SUMOylation. As_2_O_3_-induced sumoylated PML are targeted for ubiquitination by the E3 ligase RNF4 prior to the proteasome-mediated degradation (Lallemand-Breitenbach et al., 2008; Tatham et al., 2008). RNF4 harbors multiple SIMs in its N-terminus and a C-terminal RING-type E3 ligase domain. Thus, As_2_O_3_-induced, SUMO2 conjugation-dependent and ubiquitination-mediated degradation of PML depends on the binding of the SIMs in RNF4 to sumoylated PML (Lallemand-Breitenbach et al., 2008; Tatham et al., 2008). SUMOylated PML also primes casein kinase 2 (CK2)-mediated phosphorylation of PML, which also contributes to ubiquitination-mediated PML degradation as shown in APL cells, non-small cell lung carcinoma cells and human primary tumor specimens (Rabellino et al., 2012). These elegant studies of SUMOylation-mediated PML degradation authenticate the essential biological functions of the post-translational modifications of PML. Understanding such biochemical processes shall foster development of a better medicine for cancer treatment. A similar mechanism was adopted by another SIM-containing protein, ORF61, which contains an N-terminal RING-type E3 ligase domain and a C-terminal SIM motif, both of which are required for PML degradation in Varicella-zoster virus (VZV)-infected cells (Wang et al., 2011).

DNA damage also triggers PML SUMOylation as evidenced by the observation that the treatment with adriamycin, a DNA-damaging chemotherapeutic agent, increases the amount of SUMO-conjugated PML (Gresko et al., 2009). Inhibition of proteasome-degradation by MG132 treatment disrupts PML NBs and results in accumulation of PML and SUMO1 in the nucleolus where these two proteins are not normally colocalized (Mattsson et al., 2001). By contrast, heat shock causes acute de-SUMOylation of PML (Nefkens et al., 2003). Additionally, some but not all viral infections can abolish PML SUMOylation (Muller and Dejean, 1999). PML NBs undergo fission in response to stresses such as heat shock, heavy metal exposure, and expression of adenoviral protein E1A and such fission can be rescued by ectopic expression of SUMO1 (Eskiw et al., 2003). These observations raise several important questions: How do PML NBs respond to their environment through SUMOylation? What is the mechanism that senses a stress and promptly modulates PML SUMOylation? What are the consequences when PML is conjugated by different SUMOs at distinct sites?

SUMOylation of PML regulates transcription directly and indirectly, through sequestration of or dissociation of the transcription factors from PML NBs (Lehembre et al., 2001; Pearson and Pelicci, 2001; Lin et al., 2003; Gao et al., 2008a; Ohbayashi et al., 2008). For example, IL-6 treatment of cells induces the SUMO deconjugating enzyme SENP1, which in turns removes PML SUMO moieties, thereby releasing STAT3 from PML NBs and de-repressing PML-dependent STAT3 transcriptional activity (Kawasaki et al., 2003; Ohbayashi et al., 2008).

SUMOylation of PML regulates its ability to regulate apoptosis and the outcome probably depends on specific cellular contents and apoptotic stimuli. Ectopic expression of SUMO1 increases SUMOylation of nuclear PML proteins, PML NBs and protects rheumatoid arthritis synovial fibroblasts against Fas-induced apoptosis. The mechanism involves the localization of death_domain-associated protein (Daxx) to PML NBs (Meinecke et al., 2007). In addition, arsenic trioxide induces apoptosis through a PML SUMOylation-dependent pathway (Zhu et al., 1997; Lallemand-Breitenbach et al., 2008; Tatham et al., 2008; Zhang et al., 2010). One key function of PML is to protect cells from viral infection and SUMOylation also regulates PML’s anti-viral activities. Infection by poliovirus induces phosphorylation-dependent PML SUMOylation and the redistribution of PML NBs, which in turn protects p53 against virus-mediated degradation (Pampin et al., 2006). On the other hand, SUMOylation of PML can be the Achilles’ heel that attracts certain viral proteins to attack PML NBs. During human herpesvirus (HSV-1) infection, viral protein ICP0 binds to sumoylated PML through its own viral SIM motif (Boutell et al., 2011) and mediates the redistribution and disruption of PML NBs (Everett and Maul, 1994; Maul and Everett, 1994), resulting in proteasome-degradation of PML (Everett et al., 1998; Boutell et al., 2011). These observations suggest that SUMOylation of PML may be required, but not sufficient to dictate the biological outcomes of these stimuli. These distinct activities are likely determined by detailed information on which residues in PML are modified by which SUMO moiety as well as events other than PML SUMOylation.

The regulators that modulate the extent of PML SUMOylation include E3 SUMO ligases and non-E3 proteins. PML SUMO E3 ligases bind both PML and the sole E2 enzyme UBC9 to facilitate SUMO conjugation. RAN binding protein 2 (RanBP2) was first identified PML SUMOylation E3 ligase (Tatham et al., 2005) and mediates the SUMOylation of PML at K490 (Satow et al., 2012; **Figure 3**). RanBP2-mediated SUMOylation of PML is required for the maintenance of PML NBs (Saitoh et al., 2006). Recently, the protein inhibitor of activated STAT 1 (PIAS1), a well-studied SUMO E3 ligase has also been proposed as a PML SUMO E3 ligase that promotes SUMOylation of K65 and K160, which facilitates CK2-mediated, phosphorylation-dependent PML degradation (Rabellino et al., 2012). Gao et al. (2008b) demonstrated that histone deacetylase 7 (HDAC7) is required to maintain PML SUMOylation and PML NBs, but it remains unclear whether HDAC7 is an E3 ligase. Beta-catenin, a protein whose gene is highly mutated in colorectal carcinomas, has recently been shown to inhibit RanBP2-mediated SUMOylation of PML by inhibiting the interaction between RanBP2 and PML (Satow et al., 2012). The NAD-dependent deacetylase sirtuin-1 (Sirt1) also promotes PML SUMOylation independent of its deacetylase activity (Campagna et al., 2011). Intuitively, both positive and negative regulators must exist to control PML SUMOylation. Even a single modulator could regulate PML SUMOylation bilaterally in response to different cellular signals. An intriguing question is how the PML–UBC9 complex directs these different modulators. Since PML SUMOylation is highly responsive to numerous stimuli, other cofactors that transduce signals to UBC9 may participate in its regulation. Future studies will dissect the spatial–temporal regulation of PML SUMOylation as PML is likely to encounter a different spectrum of E3 enzymes, depending upon its localization.

The SUMO-specific protease (SENP) family proteins are the only proteases identified to date that specifically de-conjugate SUMO moieties from target proteins. Among the six SENPs, SENP1, -2, -3, -5, and -6 have been shown to remove SUMO conjugation of PML. SENP1 de-conjugates sumoylated PML but not RanGAP1, another sumoylated protein (Gong et al., 2000). An isoform of SENP2, SuPr-1, activates c-Jun transactivation activity through the removal of a SUMO1 moiety from PML (Best et al., 2002). SENP3 is activated by mild oxidative stress and de-conjugates PML poly SUMO2/3 chains, thereby disrupting PML NBs, promoting cell proliferation, and increasing the growth of xenografted tumors (Han et al., 2010). SENP5, localized in nucleoli, preferentially de-conjugates poly SUMO2/3 chains at K160 and K490; while removing all three SUMO paralogs at K65 (Gong and Yeh, 2006; **Figure 3**). SENP6 (aka. SUSP1) shows specificity for SUMO2/3 but not SUMO1-conjugated PML (Mukhopadhyay et al., 2006; Hattersley et al., 2011). Loss of SENP6 results in the accumulation of SUMO2/3 in PML NBs, an increase in number and size of PML NBs and a decrease in cell viability (Mukhopadhyay et al., 2006; Hattersley et al., 2011). How SENPs regulates PML NBs is an intriguing and incompletely understood question but is tied to the dynamics and function of PML NBs.

Several non-PML components of PML NBs are also sumoylated. The E3 SUMO ligase MMS21 is a component of the alternative lengthening telomere (ALT)-associated PML NBs. It promotes SUMOylation of several telomere binding proteins such as TRF1 and TRF2 (Potts and Yu, 2007). SUMOylation of ALT-PML NB components is an essential step for homologous recombination-mediated elongation of telomeres in ~ 25% of cancers. Additionally, SUMOylation and de-SUMOylation of the orphan nuclear receptor LRH-1 control its shuttling in and out of PML NBs (Chalkiadaki and Talianidis, 2005). Another example is the Daxx protein, an intrinsic PML NB component that is sumoylated. Through its SIM motif, Daxx interacts with the SUMO moieties of PML and this interaction directs the SUMOylation of Daxx itself (Lin et al., 2006).

## PHOSPHORYLATION OF PML

Phosphorylation is a common modification for transducing signals (Hunter, 2012). Phosphorylation of PML is a major regulatory mechanism that controls PML protein abundance and the number and size of PML NBs. Cells respond to various stimuli, in part, by modulating phosphorylation of PML. Here we reviewed phosphorylation in several regions of PML that have been linked to its biological functions. These regions include the N-terminal proline-rich region, the RBCC domain, a region containing a mapped ubiquitination site (K401; Hakli et al., 2005; Lallemand-Breitenbach et al., 2008), the nuclear localization sequence (NLS) and the C-terminal SIM (**Figure 2**).

Promyelocytic leukemia protein harbors an N-terminal proline-rich region (amino acids 3–46, proline 36%, **Figure 2**). Proline-rich peptides are usually exposed on a protein’s surface and thus participate in protein–protein interactions, signal transduction, and post-translational modification (Kay et al., 2000). Within this region, PML is phosphorylated at S8, S36, S38, S40, and T42 in response to epidermal growth factor (EGF) treatment (Olsen et al., 2006). It is likely that extracellular signal-regulated kinases (ERK1/2), an EGF downstream kinase, directly phosphorylates PML at T28, S36, S38, and S40 (**Figure 3**) and these phosphorylation promotes PML SUMOylation (Hayakawa and Privalsky, 2004). Additionally, following DNA damage, the homeodomain-interacting protein kinase 2 (HIPK2) also phosphorylates S8, S36, and S38 (Gresko et al., 2009; **Figure 3**). Such HIPK2-mediated phosphorylation leads to increased accumulation of PML protein and its SUMOylation and is required for the maximal pro-apoptotic activity of PML after DNA damage.

Multiple sites on PML are phosphorylated in response to DNA damage. The number of PML NBs increases following double strand breaks (DSBs). However, inhibition of ATM by caffeine or wortmannin markedly delays or inhibits the increase in PML NB number (Dellaire et al., 2006). This observation suggests that DNA damage-responsive kinase ATM regulates PML NB dynamics in response to DSB. Whether ATM directly phosphorylates PML or a PML NB component is not clear, nonetheless it is an intriguing question worth future investigation. Within the RBCC domain, the DNA damage check point kinase, Chk2, phosphorylates PML at S117 in response to gamma irradiation (Yang et al., 2002; **Figure 3**). Such phosphorylation is important for PML-mediated apoptosis following DNA damage. Additionally, PML is also phosphorylated by the ataxia telangiectasia and rad-3-related kinase, ATR kinase, which is required for the nucleolar localization of PML (Bernardi et al., 2004). However, the specific phosphorylation site is unclear.

Several groups have reported that regions surrounding the NLS and SIM of PML are phosphorylated in response to distinct stimuli. For example, phosphorylation of PML at S518, S527, and S530 was detected by mass spectrometry in EGF-treated HeLa cells (Olsen et al., 2006), during granulopoiesis of IL-3-dependent myeloid cells (L-G; Tagata et al., 2008) and in human Jurkat cells following CD3 activation of T-cell receptors (Mayya et al., 2009). Reineke et al. (2008) also reported that S403 and S505, in addition to S518, S527, and S530, were phosphorylated in HeLa cells and that the prolyl-isopeptidase Pin1 promotes PML degradation that is dependent on phosphorylation of these residues. Lim et al. (2011) later identified ERK2-dependent phosphorylation of PML at S403 and S505, an event that promotes Pin1-mediated PML degradation (**Figure 3**). Additionally, a recent study using a prostate cancer model showed that the CDK1/2-mediated phosphorylation of PML at S518 and the subsequently Pin1-mediated isomerization of PML at the S518-P519 motif facilitate Cullin3-KLHL20 ubiquitin ligase-dependent degradation of PML under the tumor hypoxia conditions (Yuan et al., 2011). Such post-translational modification-mediated degradation of PML is an essential component in HIF1α-mediated tumor hypoxia responses (Yuan et al., 2011). Interestingly, S403 is close to the ubiquitination site K401 while S505 and S518 are adjacent to the acetylation site K515, raising the possibility of mutual regulation of these modifications. The mitogen-activated protein kinase (MAPK) BMK1/ERK5 phosphorylates PML at S403 and T409 and inhibits PML-dependent activation of p21 expression (Yang et al., 2010; **Figure 3**). ERK1/2 also phosphorylates PML at S527 and S530, and is involved in As_2_O_3_-induced PML-mediated apoptosis (Hayakawa and Privalsky, 2004). Interestingly, SUMOylation of PML at three canonical SUMOylation sites (K65, K160, and K490) does not seem to be required for phosphorylation of PML at S505, S518, S527, and S530 (Tagata et al., 2008). However, PML phosphorylation deficiency at these four serines leads to slower-migrating SUMOylation bands among which certain sumoylated PML species accumulate (Tagata et al., 2008). These observations suggest that phosphorylation modulates SUMOylation in this region. PML is a key cell cycle regulator. Overexpression of PML arrests HeLa cells at G1/S (Mu et al., 1997), while loss of PML promotes cell cycle progression (Wang et al., 1998). Therefore, it is not surprising that phosphorylation of PML may be subjected to cell cycle regulation. In HeLa cells, PML directly interacts with Aurora Kinase A (AURKA) and is phosphorylated at S403, T409, S518, S527, and S530 during M phase with modest phosphorylation at S527 and S535 during the G1 phase (Dephoure et al., 2008). AURKA may participate in these phosphorylation events during the cell cycle but participation by other kinases is also possible. It is also unclear whether phosphorylation of PML at these sites plays a role in cell cycle control.

Casein kinase 2 phosphorylates PML at S565 (SSSEDSE, 560–566, **Figure 3**), which is adjacent to the SIM (VVVI, 556–559) and promotes PML degradation (Scaglioni et al., 2006). Interestingly, this CK2-mediated phosphorylation is also required for the interaction of SIM with the SUMO moiety (Stehmeier and Muller, 2009). Although it is not clear whether they are directly phosphorylated by CK2 (Scaglioni et al., 2006), the residues S560–562 are essential for SIM function (Percherancier et al., 2009; Stehmeier and Muller, 2009). Together, these studies suggest that phosphorylation of PML at regions next to its ubiquitination site, NLS and SIM are critical for PML’s function and regulation. A question that remains incompletely answered is how phosphorylation is coordinated with other post-translational modifications of PML in response to different cellular stimuli.

PML NBs are also active nuclear depots for the phosphorylation of non-PML proteins. HIPK2 kinase phosphorylates p53 at S46 in PML NBs in response to UV radiation (D’Orazi et al., 2002; Hofmann et al., 2002) and promotes subsequent p53 acetylation at K382 by CBP (Hofmann et al., 2002). Such phosphorylation and acetylation of p53 in PML NBs enhance its transactivation activity, pro-apoptotic activity, and the ability to arrest the cell cycle (D’Orazi et al., 2002; Hofmann et al., 2002; Moller et al., 2003). In addition, following DNA damage, Chk2 and p53 are enriched in PML NBs, where PML promotes Chk2 autophosphorylation, phosphorylation of p53 by Chk2 at Ser20 and subsequent stabilization of p53 (Louria-Hayon et al., 2003; Yang et al., 2006). Imatinib, a drug used to treat chronic myeloid leukemia (CML), induces TAp73 (a p53 family member) phosphorylation and localization to PML NBs in a p38- and PML-dependent manner in CML cells (Liu et al., 2009).

## UBIQUITINATION AND ACETYLATION OF PML

To date, ubiquitination of PML is only associated with its own degradation. RNF4 promotes the ubiquitination of PML at K401 and mediates As_2_O_3_-induced PML degradation through the proteasome pathway (Hakli et al., 2005; Lallemand-Breitenbach et al., 2008; Tatham et al., 2008; Weisshaar et al., 2008). Guan et al. (2012) recently identified UHRF1 as a PML ubiquitin E3 ligase, but whether K401 is target for modification and subsequent proteasome-mediated degradation is not clear. Additionally, viral proteins such as the HSV-1 viral proteins ICP0 and STUBL are E3 ligases that target PML for ubiquitination-mediated degradation in a SUMOylation-dependent manner (Boutell et al., 2003, 2011). An intriguing but unanswered question is whether PML is conjugated with ubiquitin at sites other than K401 and whether ubiquitin plays a role in signal transduction in PML NBs.

Acetylation of PML at K487 and K515 by p300 promotes its SUMOylation and is important for Trichostatin A (TSA)-induced apoptosis (Hayakawa et al., 2008). Paradoxically, overexpression of the deacetylase Sirt1 increases PML protein abundance and SUMOylation, whereas loss of Sirt1 decreases PML protein accumulation (Campagna et al., 2011). This Sirt1-dependent increase in PML protein abundance is independent of its deacetylase activity (Campagna et al., 2011), although Sirt1 promotes deacetylation of PML (Miki et al., 2012). Mutation at K487R abolishes nuclear localization of PML, but a specific role for K487 acetylation in PML nuclear localization is not clear. In contrast, a K515 acetylation deficiency in PML has minor effects on PML SUMOylation or PML NBs (Duprez et al., 1999). A further complexity was revealed by the observation that SUMOs can be acetylated. Although the acetylation on SUMO1 at K37 and SUMO2 at K33 show minor effects on PML SUMOylation, the acetylation on SUMOs appears to play an inhibitory role on PML NB assembly, through prevention of the interaction between the PML SUMO moiety and SIMs of other PML NB components, such as DAXX (Ullmann et al., 2012).

## OTHER POST-TRANSLATION MODIFICATIONS AND PML

To our knowledge, methylation of PML has not been reported. However, several protein arginine *N*-methyltransferases (PRMTs) reside in and regulate the dynamics of PML NBs (Boisvert et al., 2005; Cho et al., 2011; Neault et al., 2012). Isgylation is an ubiquitination-like post-translational modification that conjugates the interferon stimulating gene 15 (ISG15) to target proteins. Isgylation is implicated in protein synthesis, and stability control. PML is indirectly involved in isgylation as suggested by results with retinoic acid treatment that mediates degradation of PML–RARα fusion protein, in part by up-regulating the isgylation E1 enzyme UBE1L (Kitareewan et al., 2002; Pitha-Rowe et al., 2004). This UBE1L-mediated degradation of PML–RARα is believed to occur by targeting the PML domain in PML–RARα (Shah et al., 2008). Additionally, ectopic expression of an isgylation de-conjugation enzyme UBP43 leads to elevated accumulation of PML (Guo et al., 2010). These data indicate that isgylation plays a role in the regulation of PML protein accumulation. However, a mass spectrometry study did not find PML among the isgylated proteins (Giannakopoulos et al., 2005). The possibility exists that UBE1L promotes isgylation of other proteins, which in turn modulate PML protein accumulation.

## PROSPECTIVE

**FIGURE 4:**
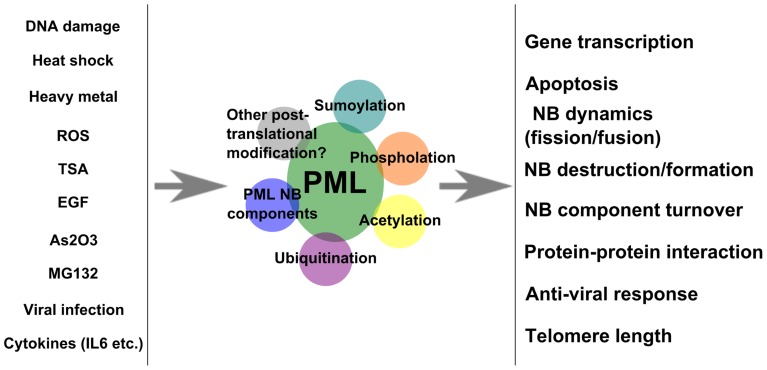
**Effects of extracellular stimuli on PML**. A model summarizes stimuli that regulate PML modification and outcomes.

Promyelocytic leukemia protein is post-translationally modified in response to different cellular stimuli (**Figure 4**). The different modifications form a complex regulatory network that modulates the activity of PML and PML NBs. It is clear that stimuli such as As_2_O_3_ and DNA damage induce both SUMOylation and phosphorylation of PML. The biggest challenge lies in the dissection of the biological effects of these modifications and the crosstalk among these modifications. The nature of low abundance or transient modification also serves as a barrier for this issue. However, the study and understanding of PML and PML NB-associated post-translational modifications will be necessary to establish its function at the molecular level.

## Conflict of Interest Statement

The authors declare that the research was conducted in the absence of any commercial or financial relationships that could be construed as a potential conflict of interest.

## Acknowledgments

We thank Dr. Samols for reading this manuscript. This work is supported by the NIH R01 HL093269, DK078965 to Hung-Ying Kao and Case Comprehensive Cancer Center Program in Aging and Energy Balance, NCI P20 CA103767.
